# Management of *Plasmodium vivax* risk and illness in travelers

**DOI:** 10.1186/s40794-017-0049-x

**Published:** 2017-03-28

**Authors:** J. Kevin Baird

**Affiliations:** 1 Eijkman-Oxford Clinical Research Unit, Jalan Diponegoro No.69, Jakarta, 10430 Indonesia; 2 Centre for Tropical Medicine & Global Health, Nuffield Department of Medicine, University of Oxford, Oxford, UK

**Keywords:** *Plasmodium vivax*, Malaria, Therapy, Prevention, Chemoprophylaxis, Primaquine

## Abstract

Malaria poses an exceptionally complex problem for providers of travel medicine services. Perceived high risk of exposure during travel typically prompts prescribing protective antimalarial drugs. Suppressive chemoprophylactic agents have dominated strategy for that practice for over 70 years. This broad class of therapeutic agents kills parasites after they emerge from the liver and attempt development in red blood cells. The dominance of suppressive chemoprophylaxis in travel medicine stems largely from the view of *Plasmodium falciparum* as the utmost threat to the patient – these drugs are poorly suited to preventing *Plasmodium vivax* and *Plasmodium ovale* due to inactivity against the latent liver stages of these species not produced by *P. falciparum*. Those hypnozoites awaken to cause multiple clinical attacks called relapses in the months following infection. Causal prophylactic agents kill parasites as they attempt development in hepatic cells. The only drug proven effective for causal prophylaxis against *P. vivax* is primaquine. That drug is not widely recommended for primary prophylaxis for travelers despite preventing both primary attacks of all the plasmodia and relapses of *P. vivax*. The long-held perception of *P. vivax* as causing a benign malaria in part explains the dominance of suppressive chemoprophylaxis strategies poorly suited to its prevention. Recent evidence from both travelers and patients hospitalized in endemic areas reveals *P. vivax* as a pernicious clinical threat capable of progression to severe disease syndromes associated with fatal outcomes. Effective prevention of clinical attacks of vivax malaria following exposure during travel requires primary causal prophylaxis or post-travel presumptive anti-relapse therapy following suppressive prophylaxis.

## Global risk

The malaria caused by *Plasmodium vivax* occurs wherever there is *Plasmodium falciparum* with few exceptions (e.g., Haiti), and sometime occurs as the sole malaria present (e.g., the Korean Peninsula). Although much of Africa has been thought virtually free of *P. vivax*, it is nonetheless relatively common all along the northern Sahel, the Horn, and on Madagascar [1], and travelers to central, western, and southern sub-Saharan Africa acquire *P. vivax* despite dominance of supposedly protective erythrocyte surface Duffy factor negativity in those populations [1, 2]. Among 618 cases of *P. vivax* in European travelers reported between 1999 and 2003, 21% were acquired in western, central and southern areas of Africa where *P. vivax* is not prevalent [1, 2]. Likewise, among 176 repatriated patients diagnosed with *P. vivax,* 32% acquired the infection in sub-Saharan Africa [3]. Recent evidence suggests *P. vivax* may utilize erythrocyte surface molecules for invasion other than Duffy factor [4]. Transmission without detectable prevalence was suggested by the finding of 13% of residents of the Congo in west central Africa being positive for antibodies specific to *P. vivax* sporozoites [5]. About 2.8 billion people live at risk of *P. vivax* with the majority of infections occurring in South and Southeast Asia [6]. This species dominates as the cause of most malaria infections in endemic Central and South America. Virtually everywhere malaria is transmitted, *P. vivax* is present [7] and must be considered in advice to patients visiting those areas.

## Virulence

Appropriate caution demands awareness of *P. vivax* as an often-inherently virulent parasite capable of causing severe malaria syndromes associated with death [8]. Although virulence is known to vary with *P. vivax* strains, none occurring today have been demonstrated to rarely progress to severe states of illness. In hospital-based studies from all across the globe, dangerous virulence appears to be common, with severe anemia and thrombocytopenia, respiratory distress, hepatic dysfunction, kidney injury and renal failure, seizures and coma, and circulatory collapse sometime occurring with acute *P. vivax* malaria [9]. During the 1920s and 1930s, patients with neurosyphilis were treated with induced attacks of *P. vivax* malaria, and 5–15% of otherwise relatively healthy patients did not survive those attacks, most dying at the sixth paroxysm [8]. A meta-analysis of risk of defined severe malaria syndromes among patients living in endemic areas and admitted to hospital with a primary diagnosis of vivax or falciparum malaria showed equal risk between these species (OR = 0.94; 95%CI, 0.2–4.4) [10]. Similar analyses showed slightly lower risk of death with vivax relative to falciparum malaria (OR = 0.64; 95%CI, 0.5–0.8) among hospitalized patients [11]. Among patients admitted to American hospitals, those with a diagnosis of *P. falciparum* were more likely to be classified as severely ill relative to *P. vivax* (OR = 7.7; 6.3–8.8), but among the severely ill risk of death between the species did not differ significantly (OR = 1.6; 0.8–3.2) [12]. These findings also emerged with *Plasmodium ovale* malaria for severe illness (OR = 5.0; 3.1–8.0) and death with severe illness (OR = 0.84; 0.2–3.7) relative to *P. falciparum* among those patients treated in the United States [12]. Patients diagnosed with *P. vivax* or *P. ovale* should be considered at risk of progression to severe disease syndromes as dangerous as those associated with a diagnosis of *P. falciparum*.

## The relapse problem

A single infectious bite by an anopheline mosquito bearing sporozoites of *P. falciparum* results in a single attack of malaria, whereas the same bearing *P. vivax* often yields five or more attacks within a year or two. The risk, timing, and number of relapses vary according to origin of infection [13]. Temperate strains and those from the Indian sub-continent are typically less likely to relapse (about 30%) and do so about 6–8 months following infection, but then also cause multiple attacks (3 may be typical). Strains of *P. vivax* from Southeast Asia and Oceania very often (>80% of patients) relapse within 3 weeks of patency of the primary attack and with multiple attacks to follow at approximately 2-month intervals [14]. In two cohorts of Indonesian travelers totaling 1182 people, 382 suffered *P. vivax* attacks within 6 months after return from travel; 80 of those were not treated with primaquine and 79% relapsed, most within 3 months of patency [15, 16]. Among 207 *P. vivax* patients in Thailand treated with chloroquine alone, 79% had recurrent parasitemia within 2 months [17]. At least some South American strains of *P. vivax* behave similarly [18]. Schwartz and colleagues [19] evaluated 300 cases of malaria among Israeli travelers, and 134 (45%) occurred more than 2 months following return from travel, with *P. vivax* contributing 129 (96%) of those attacks. Those authors considered 81% of those patients as having followed their suppressive chemoprophylaxis regimens in accordance with national guidelines. Travelers provided suppressive chemoprophylaxis and exposed to *P. vivax* but not treated with primaquine at termination of travel face high risk of clinical attacks [20].

## Suppressive chemoprophylaxis, PART and G6PD deficiency

Suppressive chemoprophylaxis does not impact the latent liver forms of *P. vivax*. Protection against late attacks requires presumptive anti-relapse therapy (PART) with primaquine (0.5 mg/kg daily for 14 days) after travel. Not all authoritative guidelines recommend PART after travel under suppressive chemoprophylaxis [21–27] and some recommend against it [24]. Among those that do recommend post-travel PART, it is reserved for patients exposed to high risk of *P. vivax* for relatively prolonged periods [21, 26]. The apparent reluctance to recommend prescribing PART may stem from the relative difficulty of safely and effectively doing so. Some view the 14 daily doses as onerous, and primaquine causes a potentially threatening hemolytic anemia in patients having an inborn deficiency of the enzyme glucose-6-phosphate dehydrogenase (G6PD) [28] necessitating laboratory screening time and costs.

Safe administration of this regimen of primaquine indeed demands affirming G6PD normal status, and adherence to the two weeks of dosing may be viewed as inconvenient or improbable. Nonetheless, if patients and providers alike understand the potentially threatening nature of acute attacks by *P. vivax*, G6PD screening and good adherence may be viewed as less problematic than with a supposedly benign and not threatening illness. Further, recently available point-of-care G6PD diagnostics may ease the inconvenience and cost of that screening [29]. The U.S. Food and Drug Administration approved at least one such screening kit [30].

Users of qualitative G6PD screening kits of any type should be aware of the insensitivity to G6PD levels above 30% of normal enzyme activity [31]. This often occurs among female heterozygotes for this X-linked trait having red blood cell populations mosaic for the deficiency at any frequency between 0 and 100% due to the phenomenon of lyonization during embryonic development. Females screened as “normal” may thus be at risk of up to 70% hemolytic loss of red blood cells with primaquine dosing [11]. Female travelers may require a quantitative laboratory assessment of G6PD status. There is no firmly established quantitative cut off of G6PD activity for safety with primaquine, but >70% of normal would exclude most heterozygotes and be unlikely to seriously threaten those included.

## Causal chemoprophylaxis

One of the most widely prescribed drugs for malaria chemoprophylaxis is Malarone® (GlaxoSmithKline UK, atovaquone-proguanil). The atovaquone component of this formulation showed causal prophylactic activity against *P. falciparum*, i.e., killed developing liver stages [32]. However, this activity may be absent or weak against *P. vivax* liver stages [33, 34], although some evidence suggests otherwise [35]. This important unresolved question requires clarification. Primaquine is the only available drug proven to arrest the development of both primary tissue schizonts and hypnozoites [36]. When taken as 0.5 mg/kg daily during exposure to infectious anophelines, primaquine prevented primary attacks of both *P. falciparum* and *P. vivax* with good efficacy (>90%), in addition to preventing the formation of hypnozoites of *P. vivax* [36, 37].

Confusion regarding the term causal prophylaxis merits some clarification here. Some authorities explain that causal prophylaxis impacts only developing tissue schizonts in the liver and does not impact hypnozoites [24]. While it is true that some drugs can prevent the formation of hypnozoites while being unable to kill formed hypnozoites [38], in practice causal prophylaxis begins in travelers free of infection. In the specific instance of primaquine as causal prophylaxis, it prevents the formation of both tissue schizonts and hypnozoites, like the experimental drug PI4 kinase [38]. It is thus incorrect to infer that causal prophylactics will not prevent the seeding of the liver with hypnozoites. Primaquine prophylaxis, unlike PI4 kinase [38], also happens to kill formed hypnozoites in patients infected without causal prophylaxis, i.e., as post-suppressive prophylaxis PART or radical cure of acute vivax malaria.

Primaquine as primary causal prophylaxis offers several distinct advantages. No pre-travel dosing is necessary, and post-travel dosing of a few days suffices. The regimen of course also abrogates the need for post-travel PART. These attributes give primaquine chemoprophylaxis great appeal for travelers departing on short notice or only briefly exposed to risk (e.g., a week or so) whom may otherwise endure several weeks of pre- and post-travel dosing with a suppressive chemoprophylactic and PART. Nonetheless, if *P. vivax* indeed occurs wherever there is malaria risk, and the clinical necessity of preventing relapses may be accepted, then primaquine as primary prophylaxis may be considered as a viable option for any traveler, regardless of destination or duration of exposure. The clinical trials of primaquine prophylaxis dosed subjects daily for 3 to 12 months with good safety and tolerance (when administered with a snack or meal) and efficacy against *P. vivax* and *P. falciparum* [36].

The principal shortcomings with primaquine for primary prophylaxis include the necessity of daily dosing, G6PD screening, contraindications with pregnancy or infancy, and emerging evidence of cytochrome P-450 isozyme 2D6 (CYP2D6) polymorphisms rendering primaquine inactive in some patients [39, 40]. The frequency of CYP2D6 alleles associated with therapeutic failure of primaquine is not known and varies widely among ethnic populations, but 1–10% may be a reasoned forecast [41]. Complete assurance of primaquine activity may require ascertaining CYP2D6 genotype, a relatively expensive and sophisticated laboratory analysis with a complex and nuanced clinical interpretation. Another potential pitfall with primaquine is its lack of activity against the blood stages of *P. falciparum*, despite good activity against the same of *P. vivax*. The traveller experiencing a breakthrough of *P. falciparum* may not be protected by on-going primaquine prophylaxis, whereas a suppressive prophylactic regimen would perhaps slow progression of the infection despite failure. Finally, the label of primaquine does not include an indication for primary prophylaxis, necessitating off-label prescribing.

Travel medicine experts informing guidance on malaria chemoprophylaxis have not put primaquine as primary causal prophylaxis forward as a recommended option except as a last resort or for special circumstances (Table 1). Some of the pitfalls listed above likely weighed in that deliberation. Acknowledging that no regimen of chemoprophylaxis is without risk and potential pitfalls, the provider weighs the relevant risks and benefits to each traveler in deciding upon appropriate protection. That weighing should include primary attacks and relapses of *P. vivax* as a potentially serious threat to travelers visiting almost any malarious area. Vivax malaria should not be considered a benign and inconsequential risk. Primaquine as primary causal prophylaxis or as PART following suppressive prophylaxis effectively deals with the problem of *P. vivax* relapses after travel. Current guidelines deal poorly with preventing those by the uniform primacy of suppressive chemoprophylaxis strategy and weakness of recommendations regarding post-travel PART in that strategy.

**Table 1:** Authoritative recommendations regarding primaquine for the prevention and treatment of *P. vivax* malaria

	United States		United Kingdom	Canada	International
Source	U.S. government	The Medical Letter	U.K. government	Canadian government	World Health Organization
Primaquine as Primary Prophylaxis	As an alternative to suppressive options or: With travel where malaria is primarily *P. vivax* Short-term travel Short-notice travel	Only as an alternative to suppressive options	Not recommended	Only as an alternative to suppressive options	None specified except for special risk groups (infants, pregnant women, vulnerable children in seasonal high transmission)
Primaquine following a diagnosis of *P. vivax*	Recommended	Recommended	Recommended	Recommended	Strongly recommended
Primaquine following suppressive prophylaxis	Recommended	No recommendation	Not recommended	Recommended for high risk groups	No recommendation
References	[21]	[22, 23]	[24, 25]	[26]	[27]

## Ideal chemoprophylaxis

The ideal drug for preventing malaria in travelers would exert both causal and suppressive activity with weekly or longer interval dosing, not threaten G6PD deficient or pregnant patients, not be reliant upon appropriate cytochrome P-450 phenotypes for good activity, and include the indication in its approved label. No such drug is currently available, although one in clinical development may more closely approach this ideal than any others now available. Tafenoquine is a primaquine-like 8-aminoquinoline under development by GlaxoSmithKline (GSK, UK) and the Medicines for Malaria Venture (MMV, Geneva) for radical cure of *P. vivax* [42]. Earlier, the drug had been under development by GSK and the U.S. Army as a chemoprophylactic agent with good efficacy against *P. falciparum* and *P. vivax* when administered monthly for up to 5 months [43]. Onset of a mild reversible vortex keratopathy occurred in most subjects of another trial dosed weekly for 6 months [44]. Tafenoquine shares the hemolytic toxicity problem of primaquine [45] and its single dose with relatively long elimination half-life elevates the importance of robust G6PD screening in conjunction with intended clinical use. Dependence upon CYP2D6 phenotypes for activity is not yet known for tafenoquine. Despite these known or potential pitfalls, should the development of tafenoquine for chemoprophylaxis resume and result in a labeled indication, it may well offer a superior option for travelers exposed to *P. vivax* and *P. falciparum* malarias.

## Radical cure of *Plasmodium vivax*

Patients diagnosed with malaria caused by *P. vivax* require therapy that terminates the acute attack (blood schizontocidal) and prevents subsequent attacks by relapse (hypnozoitocidal). This treatment is called radical cure and several options for it may be considered [46]. Many different regimens of primaquine therapy have been recommended over the 60 years of its availability as the only hypnozoitocide, but the 0.5 mg/kg daily for 14 days is now widely considered preferred among those [27, 47]. Many nations recommend 0.25 mg/kg daily for 14 days, but likely do so despite relatively low efficacy [48] in order to mitigate risk of harm due to poor access to G6PD screening. Chloroquine-resistant *P. vivax* occurs globally but is most common in areas of Southeast Asia and Oceania [49]. Treatment of those infections necessitates use of quinine or artemisinin-combined therapies (ACTs) with primaquine for radical cure.

Although some authorities include a recommendation for artemether-lumefantrine with primaquine for radical cure of chloroquine-resistant *P. vivax*, only dihydroartemisinin-piperaquine and artesunate-pyronaridine combined with primaquine have been demonstrated as safe, well tolerated and efficacious for radical cure in G6PD-normal non-pregnant subjects [15, 16]. The dependence of primaquine activity on CYP2D6 metabolism renders it vulnerable to impeded efficacy by partner drugs that may inhibit CYP2D6 activity. Lumefantrine happens to be a moderately potent CYP2D6 inhibitor and The Medical Letter [23] explicitly warns against use of artemether-lumefantrine with drugs that are metabolized by CYP2D6.

Evidence-based medical practice demands proof of safety, tolerability, and efficacy with drugs used in tandem, as in radical cure of *P. vivax* malaria. Blood schizontocides may not be responsibly combined with primaquine without such evidence [50]. The same will be true the hypnozoitocide tafenoquine following its aspired availability in practice. A single dose of tafenoquine combined with chloroquine for radical cure has shown good safety, tolerability, and efficacy in G6PD-normal non-pregnant research subjects infected by *P. vivax* [42]. The label of tafenoquine would thus be very likely to narrowly specify chloroquine as the partner blood schizontocide. Given the rise of chloroquine-resistant *P. vivax*, tafenoquine would inevitably be combined with artemisinin-combined therapies. Doing so responsibly will require demonstrations of safety, tolerability, and efficacy for each partner blood schizontocide in radical cure.

## Conclusions

Malaria caused by *P. vivax* occurs wherever malaria is transmitted with very few exceptions. Despite long being perceived as an intrinsically benign species, evidence now affirms this species as capable of progressing to severe disease syndromes associated with fatal outcomes, including in travelers. Effective prevention of *P. vivax* in travelers is not achieved with the suppressive chemoprophylaxis strategies that dominate authoritative guidance on preventing malaria in travelers. The latent hypnozoites of this species are not affected by suppressive chemoprophylaxis, and unless the exposed traveler is prescribed post-exposure PART with primaquine, late attacks by *P. vivax* may occur. Primary causal chemoprophylaxis using daily primaquine (0.5 mg/kg) during exposure prevents primary attacks of *P. falciparum* and *P. vivax*, and prevents relapses by the latter with good safety, tolerability, and efficacy in G6PD normal non-pregnant subjects (>90%), despite likely failures due to relatively infrequent CYP2D6 inadequate metabolizer phenotypes. Providers of travel medicine services should consider the advantages and pitfalls of both suppressive and causal prophylactic strategies in preventing attacks of *P. vivax* now firmly linked to risk of poor clinical outcomes. Both strategies apply primaquine, either as PART or primary prophylaxis, respectively, and necessitates coping with risk to G6PD-deficient or pregnant patients. Likewise, travelers suffering acute attacks of *P. vivax* should be considered at high risk of multiple recurrences if they do not receive radical cure employing primaquine, the only therapeutic option at present. In all facets of responsibly managing risk and illness of *P. vivax* in travelers, primaquine is involved and providers have to deal with the serious problem of its toxicity with G6PD deficiency. This will remain especially true following the anticipated registration and availability of tafenoquine as a single dose (with chloroquine) agent for radical cure of *P. vivax* malaria.

## Abbreviations

ACT: Artemisinin combined therapy
CYP2D6: Cytochrome P-450 isozyme 2D6
G6PD: Glucose-6-phosphate dehydrogenase
PART: Presumptive anti-relapse treatment
